# From animal models to patients: the role of placental microRNAs, miR-210, miR-126, and miR-148a/152 in preeclampsia

**DOI:** 10.1042/CS20200023

**Published:** 2020-04-27

**Authors:** Sonya Frazier, Martin W. McBride, Helen Mulvana, Delyth Graham

**Affiliations:** 1 Institute of Cardiovascular and Medical Sciences, University of Glasgow, Glasgow, U.K.; 2 Biomedical Engineering, University of Strathclyde, Glasgow, U.K.

**Keywords:** microRNA, placenta, preeclampsia, pregnancy

## Abstract

Placental microRNAs (miRNAs) regulate the placental transcriptome and play a pathological role in preeclampsia (PE), a hypertensive disorder of pregnancy. Three PE rodent model studies explored the role of placental miRNAs, miR-210, miR-126, and miR-148/152 respectively, by examining expression of the miRNAs, their inducers, and potential gene targets. This review evaluates the role of miR-210, miR-126, and miR-148/152 in PE by comparing findings from the three rodent model studies with *in vitro* studies, other animal models, and preeclamptic patients to provide comprehensive insight into genetic components and pathological processes in the placenta contributing to PE. The majority of studies demonstrate miR-210 is upregulated in PE in part driven by HIF-1α and NF-κBp50, stimulated by hypoxia and/or immune-mediated processes. Elevated miR-210 may contribute to PE via inhibiting anti-inflammatory Th2-cytokines. Studies report an up- and downregulation of miR-126, arguably reflecting differences in expression between cell types and its multifunctional capacity. MiR-126 may play a pro-angiogenic role by mediating the PI3K-Akt pathway. Most studies report miR-148/152 family members are upregulated in PE. Evidence suggests they may inhibit DNA methylation of genes involved in metabolic and inflammatory pathways. Given the genetic heterogeneity of PE, it is unlikely that a single placental miRNA is a suitable therapeutic target for all patients. Investigating miRNAs in PE subtypes in patients and animal models may represent a more appropriate approach going forward. Developing methods for targeting placental miRNAs and specific placental cell types remains crucial for research seeking to target placental miRNAs as a novel treatment for PE.

## Introduction

Preeclampsia (PE) is a hypertensive disorder of pregnancy, with a global estimated incidence of 3–8% [1]. Although its incidence varies substantially around the world, it remains a leading cause of maternal and perinatal morbidity and mortality [2]. PE is defined by the presence of new hypertension and new-onset significant proteinuria or other maternal organ/utero-placental dysfunction occurring at or after 20 weeks of pregnancy [3]. An effective treatment that leads to resolution of maternal complications is delivery of the fetus and placenta, contributing to the body of evidence that the placenta is a necessary and central component of the pathogenesis of PE. As a complex genetic disorder, PE arises from the interaction of environmental and genetic factors, with genetic effects contributing an estimated 50% to its etiology [4,5]. In attempt to identify potential biomarker candidates and elucidate the molecular mechanisms of PE, a host of genome-wide expression profiling studies has identified hundreds of differentially expressed genes in the placentas of preeclamptic patients, highlighting the polygenic nature of PE and essential involvement of the placenta in its pathology [6,7]. Alongside these findings, a number of studies have identified altered microRNA (miRNA) expression profiles in the placentas of patients with PE [8]. In turn, mounting evidence suggests miRNAs precisely regulate the placental transcriptome and alterations in miRNA expression play a key role in the development of PE.

## MicroRNAs in preeclampsia

MiRNAs are a class of short, non-coding RNA molecules, approximately 22 nucleotides in length, involved in post-transcriptional regulation of gene expression [9]. Single-stranded mature miRNAs harbor a highly conserved sequence, known as the seed region, which consists of 2–8 nucleotides. The seed region binds via full or partial complementary base pairing to the 3′ untranslated region of messenger RNA (mRNA) of one to hundreds of target genes, inducing translational inhibition or degradation of mRNA [9]. There are now over 2600 miRNAs annotated in the human genome (miRBase v22), and it is estimated that 30–60% of protein coding genes may be subject to miRNA regulation [10,11]. A considerable number of miRNAs are conserved across species [12], indicating their involvement in essential physiological processes. Inactivation of miRNA machinery *in vivo* induces placental malformation [13] and knockdown of miRNA machinery in *ex vivo* placental explants leads to aberrant trophoblast proliferation [14], showing the critical role of miRNAs in placental development. Inhibition and overexpression of miRNAs in primary trophoblasts and trophoblast and endothelial cell lines have further demonstrated the ability of miRNAs to modulate placental development and function [15]. In addition, both rodents and primates possess species-specific miRNA clusters that are expressed primarily or exclusively in the placenta and are essential for placental and fetal development [16]. For example, knockout (KO) of the rodent-specific chromosome 2 microRNA cluster in mice leads to severely impaired placental development, embryolethality, and fetal defects [17]. Moreover, members of the primate-specific chromosome 19 miRNA cluster are significantly differentially expressed in preeclamptic patients [18,19], potential biomarkers for PE [20,21], and involved in trophoblast function through modulation of target genes [22,23]. Hence, species-specific placental miRNAs are involved in PE. MiRNAs conserved across species are also dysregulated in the placentas of patients with PE, and *in vitro* investigations have begun to elucidate the pathological pathways and downstream targets of conserved miRNAs [24–26]. However, studies examining the role of miRNAs in animal models of PE are limited, with only three studies identified in the literature that investigate the role of miR-210, miR-126, and miR-148/152, respectively. Preclinical animal models allow molecular and functional analyses of the disease mechanism not possible in humans and are therefore critical for understanding the role of placental miRNAs in the pathology of PE. Furthermore, comparing the miRNA expression profiles of animal models of PE to that of patients with PE permits evaluation of miRNAs as potential targets for novel treatments. This is particularly relevant given the inconsistency across clinical studies as to which miRNAs are differentially expressed in the placentas of preeclamptic patients and their direction of expression, which may in part be attributed to patient characteristics (such as ethnicity, gestational age, presence or absence of labor, and preterm or term delivery) and differences in experimental methodologies. Hence, animal models provide crucial insight into the miRNAs modulating altered gene expression in the placenta in PE and the pathological mechanisms arising from as well as governing their dysregulation.

## Rodent models of preeclampsia

Rodents are valuable animal models for studying the genetics underlying the human placenta in health and disease. The placentas of humans and rodents fall under the same classifications of discoid (referring to its gross morphology) and hemochorial (referring to the fetal epithelium bathing in maternal blood). In addition to commonalities in placental structure and function [27], genome-wide gene expression profiling suggests they share similarities in terms of placental gene expression patterns across pregnancy [28]. Rodents also undergo similar cardiovascular adaptations to those seen in human pregnancies, such as increased glomerular filtration rate and renal plasma flow [29]; reduced sensitivity to Angiotensin II (Ang II) [30]; decreased vascular tone and vasomotion [31]; and elevated cardiac output, stroke volume, and heart rate [32]. Hence, rodents have been ubiquitously utilized as animal models of PE, including through utero-placental ischemia, nitric oxide synthase inhibition, angiogenesis antagonism, inflammatory activation, and renin–angiotensin system stimulation [33]. In support of their use, rodent models commonly display the hallmark features of PE, namely hypertension and proteinuria, in addition to other PE-like symptoms, such as endothelial dysfunction, placental abnormalities, and fetal demise/growth restriction [33].

Animal models are essential to studying PE since the disorder presents almost exclusively in humans, with spontaneous PE otherwise described in only a handful of non-human primates [34–37]. The occurrence of PE in humans and non-human primates is in part attributed to extensive trophoblast invasion leading to abnormal remodeling of maternal spiral arteries that supply the placenta, a pathological process unique to these species [38]. While trophoblast invasion and maternal artery remodeling is common to humans, rats, and mice, trophoblasts invade to a notably lesser extent in rats and mice compared with humans [39]. This highlights one of the major disadvantages of rodent models, namely that PE must be induced, commonly achieved by surgical or pharmacological interventions [33]. Furthermore, differences in cell types and the ensuing mechanisms regulating placental signaling contribute to the distinct placental morphology and placental cellular functions between humans and rodents [39]. These distinguishing features in turn reflect species-specific gene expression patterns in the placenta [28]. Recapitulating the disease in rodents therefore carries limitations and extrapolating findings to humans must be carefully considered. Nonetheless, given the ethical implications of conducting research in human pregnancy, rodents represent a valuable model system for investigating the pathological mechanisms contributing to the development of PE.

## Placental microRNAs in rodent models of preeclampsia

Three studies utilizing animal models of PE were conducted in rodents to explore the role of placental miRNAs, miR-210, miR-126, and miR-148/152 respectively, by examining expression of the miRNAs, their regulators, and their gene targets [40–42]. The following review examines the role of miR-210, miR-126, and miR-148/152 in PE by comparing their direction of expression in PE animal model studies to those reported in PE patients. Moreover, cross-examining the expression of regulators and potential gene targets of miRNAs identified in the PE animal model studies with evidence from *in vitro* studies, other PE animal models, and preeclamptic patients offers comprehensive insight into the genetic components and pathological processes in the placenta that contribute to the disease etiology. Overall, preclinical and clinical studies provide unique evidence of the placental genetic factors involved in PE. Identifying similarities and differences between these studies as well as incorporating evidence from *in vitro* studies remains crucial to deepen our knowledge of the genetic pathways contributing to the pathogenesis of PE, with the potential to identify targets for novel treatments in PE.

## MiR-210

MiR-210 has been implicated in a variety of physiological processes, including cell proliferation, differentiation, metabolism, and apoptosis; cell cycle regulation; mitochondrial function; angiogenesis; neurogenesis; erythropoiesis; and spermatogenesis (reviewed by [43]). Concurrently, its aberrant expression in a range of disease states, such as tumorigenesis, cancer, status epilepticus, cryptorchidism, and cardiovascular diseases (CVDs), has prompted investigations into the role of miR-210 in different pathological processes [43]. MiR-210 is now recognized as a key hypoxia-response factor both in healthy and disease states, underlying its name as the master ‘hypoxamir’, a hypoxia-inducible miRNA [44]. The induction of miR-210 in response to hypoxia has been demonstrated in practically all primary cells and cell lines investigated [45,46], and preclinical and clinical evidence has consistently shown its upregulation in ischemic tissues and conditions [47–50]. In addition to hypoxia, oxidative stress and inflammation mediate miR-210 expression, highlighting that hypoxia-independent mechanisms contribute to the role of miR-210 in physiological and pathological processes [51,52]. Likewise, evidence from animal models used to investigate the role of miR-210 in the pathophysiological processes of PE supports the involvement of both hypoxia-dependent and -independent mechanisms, findings corroborated by preclinical *in vitro* and *in vivo* studies and clinical studies in preeclamptic patients.

Only a single PE animal model study investigating the role of miR-210 in PE was identified in the literature. The study utilized mice treated with the toll-like receptor 3 (TLR3) agonist, poly I:C (P-PIC), to induce a preeclamptic phenotype [40,53]. The study found significantly greater placental miR-210 expression in TLR3-induced PE mice as compared with wild-type (WT) mice [40]. Two further preclinical studies have provided indirect evidence of miR-210 involvement in PE through examination of the miRNA and its gene targets in models of gestational hypoxia [54,55], a valuable model for drawing inferences about PE. While gestational hypoxia is not specific to PE and there are differences in the maternal physiological responses to each, high altitude placentas represent valuable models for studying PE (reviewed by [56]). High altitude and PE produce similar molecular changes in the placenta [57], and women living at higher altitudes have an increased risk of developing PE [58–60], highlighting a common pathophysiological impact. In line with the findings from the TLR3-induced PE mouse model, a significant upregulation of miR-210 was reported in the uterine arteries of pregnant sheep residing at high altitude [55]. An increase in miR-210 in these models lies in agreement with the consistent upregulation of miR-210 seen in the placentas (16 studies) and blood (6 studies) of preeclamptic patients (Table 1) and in placentas from high-altitude pregnancies [61]. However, it remains unclear whether altered miR-210 expression is a consequence or cause of PE and a harmful or protective mechanism. Nonetheless, one study found miR-210 was upregulated as early as 12 weeks of pregnancy in preeclamptic patients [62], and another study demonstrated its utility as a potential serum biomarker [63] (Table 1).

**Table 1:** Studies demonstrating a significant change in miR-210 expression in PE patients compared with healthy pregnant women

Study	Type of study	Type of PE	Time of collection	Tissue sample	Direction of change
Zhu et al. 2009 [19]	Microarray qPCR	Severe Mild	Delivery Delivery	Placenta villi Placenta villi	Up Down
Enquobahrie et al. 2011 [64]	Microarray and qPCR	Diagnosed	Delivery	8 maternal and 8 fetal sites	Up
Ishibashi et al. 2012 [65]	RNA seq and qPCR	EO, LO, and superimposed	Delivery	Placenta	Up
Muralimanoharan et al. 2012 [66]	qPCR	Severe	Delivery	Placenta villi	Up
Zhang et al. 2012 [67]	qPCR	Mild and severe	Delivery	Plasma	Up
Anton et al. 2013 [63]	qPCR	Diagnosed	Delivery and 15–20 weeks	Serum	Up
Betoni et al. 2013 [68]	Microarray	Diagnosed	Delivery	Placenta	Up
Lalevée et al. 2014 [24]	qPCR	Severe	Delivery	Placenta	Up
Luo et al. 2014 [69]	qPCR	Severe	Delivery	Basal plate Chorionic plate	Up n.s.
Ura et al. 2014 [62]	Microarray and qPCR	Severe	12–14 weeks	Serum	Up
Weedon-Fekjær et al. 2014 [70]	RNA seq	EO and LO	Delivery	Centrally located cotyledon	Up
Xu et al. 2014 [71]	Microarray qPCR	Severe Severe	Delivery Delivery 15–18 weeks and term	Placenta Basal plate Chorionic plate Plasma	Up Up n.s. n.s.
Jiang et al. 2015 [25]	Microarray	Severe	Delivery	8 maternal and 8 fetal sites	Up
Zhang et al. 2015 [72]	Microarray	Severe	Delivery	Basal plate	Up
Vashukova et al. 2016 [73]	RNA seq	Superimposed	Delivery	Placenta villi of 8 maternal and 8 fetal sites	Up
Zhou et al. 2016 [26]	RNA seq and qPCR	Diagnosed	Delivery	Chorionic plate from quadrants and central portion in placenta disc	Up
Adel et al. 2017 [74]	qPCR	Mild and severe	Delivery	Placenta villi	Up
Gan et al. 2017 [75]	qPCR	Diagnosed	Delivery	Serum Urine	Up n.s.
Jairajpuri et al. 2017 [76]	Microarray	Mild and severe	Delivery	Plasma	Up
Chen et al. 2019 [77]	qPCR	LO	Delivery	Full thickness placental biopsy	Up
Nejad et al. 2019 [78]	qPCR	Diagnosed	Delivery	Plasma	Up
Wang et al. 2019 [79]	qPCR	Diagnosed	Delivery	3–5 cm from umbilical cord attachment site (chorionic side, full thickness)	Up

EO, early onset; LO, late onset; n.s., not significant; wks, weeks. Time of collection designated ‘delivery’ if not stated in study.

In contrast with the multitude of studies reporting an upregulation, a reduction in miR-210 expression was noted in a study of pregnant mice subject to hypoxia, although the study attributed this to moderate hypoxia exposure [54]. This finding is supported by a study in patients that found a downregulation of miR-210 in patients with mild PE (Table 1) [19]. Hence, higher levels of hypoxia above a certain threshold may drive up miR-210 expression, providing an explanation for the consistent upregulation seen in severe PE. Important to note though is that several studies in mild preeclamptic patients have also observed an increase in miR-210 expression (Table 1) [67,74,76]. Furthermore, exposure of WT and miR-210 KO mice to moderate hypoxia led to an increase in placental weight, and no histological differences were observed between the groups [40]. This demonstrates miR-210 is not essential for normal fetal placental growth under moderately hypoxic conditions. It also suggests that moderate hypoxia does not induce the harmful effects potentially associated with an upregulation of miR-210 arising from severe hypoxia.

In a further two further studies in preeclamptic patients, although a significant increase in miR-210 was seen in the basal plate, a non-significant trend toward downregulation of miR-210 was evident in the chorionic plate [69,71] (Table 1). Unlike the basal plate, which primarily comprises maternal tissue, the chorionic plate consists solely of fetal tissue, including fetal blood vessels, trophoblasts, and stroma tissue. Varying proportions of maternal and fetal tissue underlie the distinct cell types, morphology, and physiological roles of the different layers of the placenta. In turn, there are variations in gene expression between placental layers [80,81] and trophoblast cell types [82], supporting the findings of heterogeneous miRNA expression. Site-specific differences in miR-210 expression across the placenta therefore probably reflect the unique placenta genome, which consists of maternal and fetal genes and their differing contributions to each layer as well as a variety of cell types. Overall, despite the diverse genetic makeup of the placenta, the majority of preclinical and clinical studies point toward an upregulation of miR-210 in PE. While inhibiting miR-210 expression in patients with severe PE may therefore represent a potential treatment strategy, precise targeting may be required to achieve a therapeutic benefit due to varied miR-210 expression across the placenta.

## Inducers of MiR-210

**Figure 1:**
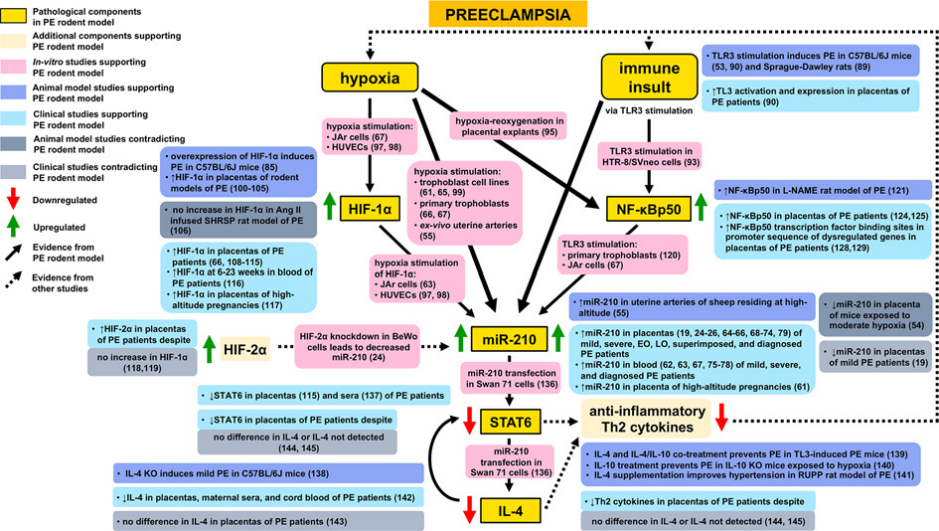
Supporting and contradicting evidence of proposed genetic components and pathological processes involved in PE based on PE rodent model study investigating the role of placental miR-210 miR-210, microRNA 210; HIF-1 α, hypoxia-inducible factor 1 α; HIF-2α, hypoxia-inducible factor 2 α; NF-κBp50, nuclear factor-κB transcriptional factor p50; STAT6, signal transducer and activator of transcription 6; IL-4, interleukin-4; IL-10, interleukin 10; Th2, T-helper type 2; PE, preeclampsia; EO, early-onset; LO, late onset; TLR3, toll-like receptor 3; HUVECs, human umbilical vein endothelial cells; RUPP, reduced uterine perfusion pressure.

To identify drivers of elevated miR-210 expression in PE, the study investigating the role of miR-210 in a TLR3-induced mouse model of PE examined the expression of transcription factors, hypoxia-inducible factor 1 (HIF-1) α and nuclear factor-κB transcriptional factor (NF-κB) p50, finding significantly increased placental expression of both factors [40]. HIF-1 is a key mediator of the cellular response to hypoxia. Under hypoxic conditions, the inducible HIF-1α subunit is stable, allowing it to form a dimer with the constitutive HIF-1β subunit and bind to hypoxia response elements (HREs) of target genes or miRNAs and regulate their expression [83]. In turn, this affects a diverse array of physiological and pathophysiological pathways (reviewed by [83]). Although HIF-1α and -2α KO mice demonstrate the essential involvement of HIFs in placental vascularization and trophoblast differentiation [84], overexpression of HIF-1 α has been shown to induce a preeclamptic phenotype in pregnant mice [85], supporting its role in the pathogenesis of PE. A growing number of studies suggest placental hypoxia modulates HIF-1α expression, mediating downstream targets involved in PE, including soluble vascular endothelial growth factor receptor-1 (sFlt-1) [86], transforming growth factor beta [87], and endoglin [88]. In addition to hypoxia, immune responses mediated by toll-like receptors may drive differential gene expression in PE. Kopriva et al. [40] found that, in TLR3-KO mice treated with the TLR3 agonist, P-PIC, the elevation of miR-210, HIF-1α, and NF-κBp50 was ameliorated, indicating an important role for TLR3 activation. In addition to stimulation of TLR3 inducing a PE phenotype in mice and rats [53,89], an upregulation of TLR3 has been observed in PE patients [90], supporting the hypothesis that excessive activation of TLR3 may play an important role in the development of PE. Double-stranded RNA, arising from virus replication or necrotic, apoptotic, or stressed cells, leads to TLR3 activation [91]. TLR3 activates two key pathways involving NF-κB and interferon regulatory factor 3, which subsequently activate pro-inflammatory cytokines and Type 1 interferons, respectively [92,93]. The NF-κB transcription factor family consists of five members divided into two subfamilies: the NF-κB proteins, NF-κB1 (p50) and NF-κB2 (p52), and Rel proteins, RelA (p65), RelB, and c-Rel, which can form homo- and heterodimers that bind to DNA target sites and induce transcription of target genes [94]. In trophoblasts exposed to the TLR3 ligand, P-PIC, and in placental explants subject to hypoxia-reoxygenation, an increase in NF-κBp50 was associated with upregulation of genes known to be involved in PE, namely the anti-angiogenic factor sFLT-1 [93], pro-inflammatory enzyme cyclooxygenase-2, and pro-inflammatory cytokines, tumor necrosis factor α (TNF-α) and interleukin 1 β (IL-1β) [95]. Dysregulation of HIF-1α and NF-κBp50 arising from hypoxia and/or immune- mediated responses and a concurrent modulation of target genes suggests the transcription factors play a key role in the pathology of PE, with miR-210 representing a potential target (Figure 1).

HIF-1α induces miR-210 expression via binding to a HRE region of the miR-210 promoter, a sequence highly conserved across species, including mice, rats, and humans [96]. *In vitro* studies have shown direct regulation of miR-210 by HIF-1α during hypoxia in trophoblastic JAr cells [67] and human umbilical vein endothelial cells (HUVECs) [97,98]. In addition, direct regulation of miR-210 by the HIF-1α isoform, HIF-2α, has been observed in the trophoblastic BeWo cell line [24]. Studies have also provided broader evidence of a hypoxia-dependent induction of miR-210 by demonstrating upregulation of the miRNA in response to hypoxia exposure in practically all primary cells and cell lines, which includes trophoblast-derived cell lines [61,65,99], primary trophoblasts [66,67], and *ex vivo* uterine arteries [55]. Collectively, these studies support that hypoxia, potentially through induction of HIF-1α, stimulates miR-210 expression (Figure 1).

PE animal models and clinical studies in preeclamptic patients reporting an increase in HIF-1α further corroborate the involvement of hypoxia in PE and explain the consistently observed upregulation of miR-210 in PE patients. Numerous PE rodent models exhibit elevated placental levels of HIF-1α [100–105]. In an Ang II infused superimposed PE rat model, there was no difference observed in placental HIF-1α in comparison with control stroke-prone spontaneously hypertensive (SHRSP) rats [106]. However, elevated levels of HIF-1α in SHRSP placentas have previously been reported, thus potentially masking increases in HIF-1α levels when used as a control [107]. Finally, overexpression of HIF-1α in C57BL/6J pregnant mice produced a PE phenotype [85]. With respect to PE patients, a plethora of clinical studies show higher protein levels of HIF-1α in third trimester placentas [66,108–115]. Significantly greater levels have also been identified in blood as early as weeks 6–23 of pregnancy [116] and in placentas from high-altitude pregnancies [117]. In studies that found no significant difference in HIF-1α levels in the placentas of PE patients, a significant upregulation of HIF-2α was noted [118,119]. These clinical findings raise the possibility that placental hypoxia plays a causal role in the development of PE, in part through upregulation of HIF-α proteins, which in turn leads to dysregulation of downstream targets, including miR-210 (Figure 1).

With regards to NF-κBp50, *in vitro* studies report the transcription factor binds to the miR-210 promoter in primary trophoblasts and JAr cells [67,120], supporting the increased NF-κBp50 and miR-210 levels seen in the placentas of the TLR3-induced PE mouse model [40]. N (gamma)-nitro-L-arginine methyl ester (L-NAME)-induced preeclamptic rats also display enhanced NF-κB activation [121] and lipopolysaccharide (LPS)-induced preeclamptic rats show elevated levels of NF-κBp65 [122,123]. In agreement with the general trend toward an upregulation of NF-κB transcriptional factors, results of immunohistochemical studies conducted in placentas from pregnancies complicated with PE show higher expression of NF-κBp50 [124,125] and NF-κBp65 [125–127]. Furthermore, *in silico* comparative promoter analyses of data from microarray studies examining altered gene expression in placentas from PE patients found a greater number of NF-κBp50 transcriptional factor binding sites in the promoter sequences of dysregulated genes [128,129]. Enhanced NF-κBp50 expression and activation in the placenta therefore probably plays a role in the pathology of PE and contributes to the upregulation of miR-210 in PE (Figure 1).

## Gene targets of MiR-210

Kopriva, et al. [40] examined gene targets of miR-210 in the PE mouse model, namely signal transducer and activator of transcription 6 (STAT6) and interleukin 4 (IL-4). IL-4 is a cytokine that plays a key role in regulating the immune system. Binding of IL-4 to its receptor activates STAT6, a mediator of T-helper type 2 (Th2) cell differentiation [130]. Activation of STAT6 by IL-4 promotes expression of GATA binding protein 3 (GATA3), a master regulator of Th2 cell differentiation, which binds and enhances expression of the anti-inflammatory Th2 cytokines [130]. An imbalance between T-helper type 1 (Th1) and Th2 cytokines is one theory underlying the pathology of PE [131,132]. Studies have found elevated levels of pro-inflammatory Th1 cytokines in preeclamptic patients in contrast with the dominant Th2 cytokine profile in healthy pregnancy [133–135]*.* Modulation of genes involved in Th2 differentiation by miR-210 provides insight into the mechanisms governing the reported shift in the immune profile of preeclamptic patients and demonstrates a role for the miRNA in the pathology of PE.

In a trophoblastic Swan 71 cell line transfected with miR-210, STAT6 was identified as a downregulated gene [136]. Previous studies investigating STAT6 expression in the placenta and maternal serum also found a reduction in STAT6 protein levels in preeclamptic patients [115,137]. This is in line with the reported downregulation of the protein levels of the miR-210 predicted target STAT6 in the placentas of TLR3-induced PE mice [40]. In addition to reduced STAT6 expression, Kopriva et al. [40] observed significantly downregulated placental IL-4 levels. In a separate study, the group showed IL-4 deficiency in mice produced a mild PE phenotype [138]. Subsequently, they found that IL-4 treatment and IL-4/ interleukin 10 (IL-10) co-treatment was able to significantly reduce blood pressure and prevent endothelial dysfunction in a TLR3-induced PE mouse model [139]. Another group utilizing IL-10 KO mice exposed to hypoxia to induce severe preeclampsia showed recombinant IL-10 restored blood pressure, proteinuria levels, and fetal weight to physiological values [140]. In reduced uterine perfusion pressure rats, another group reported IL-4 supplementation significantly improved blood pressure and normalized uterine artery resistance index [141]. Stimulating anti-inflammatory Th2 cytokines to confer therapeutic benefit in hypoxia- and immune-based preclinical models of PE suggests their downregulation, in part driven by miR-210 inhibition, may contribute to hypoxia- and immune-related pathological processes in PE. However, studies investigating IL-4 expression in placentas of PE patients have produced conflicting results. In support of the findings of Kopriva et al. [40], demonstrating a downregulation of IL-4 in the TLR3-induced PE mouse model, one study found IL-4 was lower in placental tissue, maternal serum, and cord blood in PE patients when compared with normotensive controls [142]. These findings stand in contrast to studies that showed no difference in placental IL-4 expression in preeclamptic patients [143,144] and a study in which IL-4 could not be detected [145]. However, these studies observed a decrease in other Th2 cytokines and their transcription factors [144,145]. Altogether, evidence supports a role for reduced Th2 cytokine production in PE, which may in turn be subject to miR-210 modulation (Figure 1).

## MiR-126

MiR-126 is abundantly expressed in endothelial cells (ECs) [146,147] and known to play an important role in vascular homeostasis through regulation of angiogenesis, vasculogenesis, and inflammation (reviewed by [148]). Deletion of miR-126 in mice has shown to induce embryolethality in 40–50% of cases, with embryos displaying signs of severe systemic edema and hemorrhages, and surviving adult mice exhibiting impaired angiogenesis under physiological conditions and post-injury [146,149]. In zebrafish, knockdown of miR-126 leads to abnormalities in circulation and vascular morphology [150]. Overall, this demonstrates that miR-126 plays an important role in vascular development during embryogenesis and subsequently in maintaining vascular integrity in adulthood with respect to endothelial function and post-injury repair. In further support of its protective and proangiogenic role, the downregulation of miR-126 has been reported in a number of CVDs, including atherosclerosis [151], ischemic stroke [152], heart failure [153], atrial fibrillation [153], coronary artery disease [154], and diabetes [155], with growing interest in its role as a biomarker. However, studies in different cell types, animal models, and diseases, most notably autoimmune diseases and cancer, show that miR-126 also exhibits antiangiogenic effects [156,157]. MiR-126 appears to play a number of roles often antagonistic in nature from proatherogenic [158] to antiatherogenic [159], from tumor suppressor [160] to inducer [161], and from regulator of HSC quiescence to stimulator of HSC activation [162], demonstrating its multi-regulatory capabilities within specific cell types.

**Figure 2:**
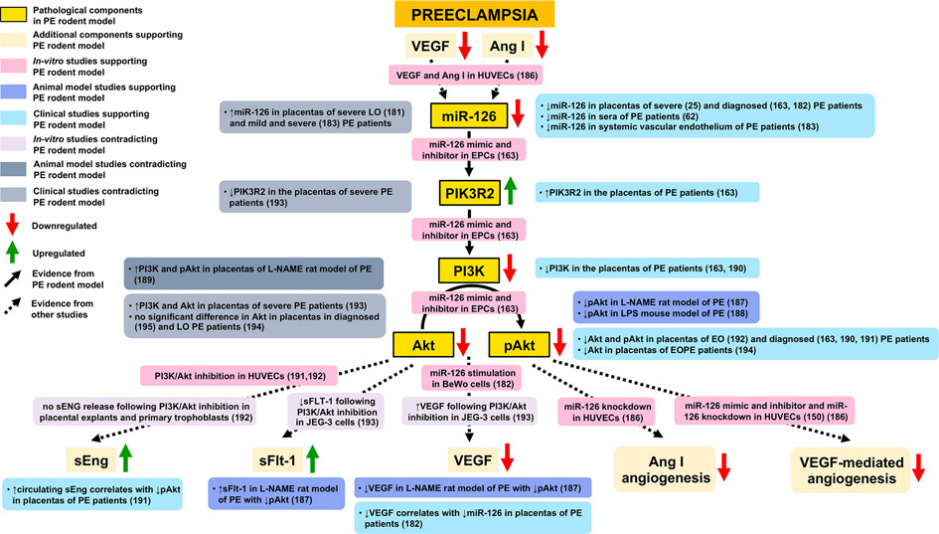
Supporting and contradicting evidence of proposed genetic components and pathological processes involved in PE based on PE rodent model study investigating the role of placental miR-126 miR-126, microRNA 126; PIK3R2, phosphatidylinositol 3-kinase regulatory subunit 2; PI3K, phosphatidylinositol 3-kinase; PI3K, phosphatidylinositol 3-kinase; Akt, protein kinase B; pAkt, phosphorylated Akt; VEGF, vascular endothelial growth factor; Ang I, Angiotensin I; sEng, soluble endoglin; sFflt-1, soluble vascular endothelial growth factor receptor-1; PE, preeclampsia; LO, late-onset; EO, early-onset; HUVECs, human umbilical vein endothelial cells; L-NAME, N (gamma)-nitro-L-arginine methyl ester; LPS, lipopolysaccharide.

The context-dependent function of miR-126 is reflected by its expression in a range of cell types besides ECs, with miR-126 identified in endothelial progenitor cells (EPCs) [163], epithelial cells [164], hematopoietic stem and progenitor cells [162], platelets [165], and several types of cancer [166–168] and immune cells [169,170]. Furthermore, the diverse roles of miR-126 in part arises from the number of factors regulating its expression. Described by some as a ‘mechanomir’, miR-126 expression has shown to be downregulated by hypoxia [171] and both stimulated [172] and inhibited [158] by laminar shear stress. Its divergent properties are also attributable to miR-126 activating the phosphatidylinositol 3-kinase-protein kinase B (PI3K-Akt) pathway, an intracellular signal transduction pathway that regulates a host of cellular processes, such as cell metabolism, proliferation, survival, growth, autophagy, and angiogenesis [173]. Phosphatidylinositol 3-kinase regulatory subunit 2 (PIK3R2) has been validated as a target of miR-126 using reporter constructs in a number of cells, including ECs [150], cancer cells [174–176], rheumatoid arthritis synovial fibroblasts [163,177], and EPCs [178,179]. Preclinical studies investigating miR-126 in the placenta suggest that miR-126 plays an important protective angiogenic role through regulation of the PI3K-Akt pathway in PE, although studies in preeclamptic patients provide conflicting results regarding its direction of expression (Figure 2).

Two studies identified in the literature have investigated the placental role of miR-126 *in vivo*. Following *in vitro* investigations in EPCs, where overexpression of miR-126 was shown to stimulate EPC proliferation, differentiation, and migration [163], Yan et al. [163] examined the effects of mimicking or silencing miR-126 in the placentas of pregnant rats. Administration of agomir-126 led to an increase in microvessel density and an increase in the size and weight of placentas and fetuses. In agreement, antagomir‐126 administration led to a reduction in microvessel density and a reduction in the size and weight of placentas and fetuses, suggesting a role for miR-126 in placental angiogenesis. These findings prompted the study that investigates the role of miR-126 in a PE animal model and examines its potential as a therapeutic target for PE [41]. Administration of L-NAME to induce a preeclamptic phenotype, demonstrated by elevated systolic blood pressure (SBP) and higher levels of urinary protein, led to a reduction in miR-126 [41]. Administration of agomir-126 led to a reduction in blood pressure, significantly greater placental and fetal weight, enhanced microvessel density, and a higher proportion of live pups, suggesting elevating levels of miR-126 in PE may represent a therapeutic approach to counteract lower levels of miR-126 in PE. However, clinical studies provide conflicting results as to the direction of miR-126 expression in the placenta in preeclamptic patients. While three studies showed a reduction in miR-126 expression in the placentas of preeclamptic patients, two studies detected an increase in expression (Table 2). These opposing findings are arguably not due to differences between subtypes of PE since miR-126 was reported as both up- and downregulated in severe PE patients. It is possible though that the contrasting results reflect differences in expression among different cell types and thus the multi-functional capacity of miR-126. Furthermore, even within subpopulations, PE patients may display diverse genetic signatures, highlighting the difficulty in deciphering the genetics of PE [180]. Despite the therapeutic benefit of increasing placental miR-126 expression in an L-NAME rat model of PE, it remains difficult to discern whether the same therapeutic benefits would translate to patients. Nonetheless, comparing data from animal models with patients provide insight into the clinical relevance of miRNAs and genes under investigation.

**Table 2:** Studies demonstrating significant change in miR-126 expression in PE patients compared with healthy pregnant women

Study	Type of study	Type of PE	Time of collection	Tissue sample	Direction of change
Hu et al. 2009 [181]	Microarray	Severe LO	Delivery	Chorionic tissue from the central part of the placenta	Up
Yan et al. 2013 [163]	qPCR	Diagnosed	Delivery	Placenta	Down
Hong et al. 2014 [182]	qPCR	Diagnosed	Delivery	Chorionic tissue from the central part of the placental maternal phase	Down
Ura et al. 2014 [62]	Microarray and qPCR	Severe	12-14 weeks	Serum	Down
Jiang et al. 2015 [25]	Microarray	Severe	Delivery	8 maternal and 8 fetal sites	Down
Yang et al. 2015 [183]	RNA seq	Mild and severe	Delivery	Placenta and plasma	Up
Xu et al. 2019 [184]	*In situ* hybridization	Diagnosed	Delivery	Systemic vascular endothelium from subcutaneous adipose tissue	Down

LO, late onset; Time of collection designated ‘delivery’ if not stated in study.

## Gene targets of MiR-126

In the study by Yan et. al [41] examining preeclamptic rats with reduced miR-126 expression, mRNA and protein levels of PIK3R2 were significantly greater while phosphorylated Akt (pAkt) and protein kinase B (PKB), also known as Akt, were significantly lower in preeclamptic rats compared with rats administered with agomir-126. There are three classes of phosphatidylinositol 3-kinase (PI3K) enzymes (class I, II, and III). Class IA enzymes consist of two domains: a catalytic and a regulatory subunit. PIK3R2 encodes the p85β regulatory subunit that represses PI3K activation by inhibiting its catalytic subunit, p110. PI3K is primarily activated by phosphorylated receptor tyrosine kinases in response to extracellular signals that abolish p85β inhibition. Activation of PI3K stimulates signal transduction pathways that lead to phosphorylation of Akt, a serine/threonine protein kinase [185]. Once activated, Akt modulates the function of a wide variety of target proteins in both the cytoplasm and nucleus and thus a broad array of cellular functions. Vascular endothelial growth factor (VEGF) mediated angiogenesis has found to be stimulated by miR-126 in endothelial cells through inhibition of sprouty related EVH1 domain containing 1 (SPRED1) and PIK3R2, which negatively regulate extracellular signal–regulated kinase (ERK) and Akt phosphorylation, respectively [146,150]. In addition, VEGF and Angiotensin I (Ang I) induction of miR-126 leading to inhibition of PIK3R2 and enhanced phosphorylation of Akt has been shown to play a role in promoting endothelial cell survival and sprouting [186]. MiR-126 has also been reported to stimulate VEGF expression in trophoblast cells, and lower levels of placental miR-126 in PE patients were associated with decreased VEGF placental expression [182]. Hence, the proangiogenic effects of miR-126 mediated via the PI3K-Akt pathway may be altered in PE (Figure 2).

In an earlier study, Yan et al. [163] utilized miRNA mimics and inhibitors to investigate PIK3R2, PI3K, Akt, and pAKT as targets of miR-126 in EPCs. A miR-126 inhibitor induced a significant increase in mRNA and protein levels of PIK3R2 and significant decrease in the levels of PI3K, pAkt, and Akt, reflecting the results observed in the L-NAME preeclamptic rat model with reduced miR-126 expression [41]. A reduction in pAkt was identified in a different study in L-NAME-induced preeclamptic rats, which found increased sFlt-1 and decreased VEGF placental expression [187]. Reduced pAkt levels were also reported in a study in LPS-induced preeclamptic mice, which displayed an increase in pro-inflammatory cytokines, TNF-α, IL-1β and interleukin-6, and placental chemokines [188]. These findings support involvement of the PI3K/Akt pathway in PE through inhibiting angiogenic and promoting inflammatory processes (Figure 2). It should be noted though that a separate study in L-NAME preeclamptic rats reported an upregulation of placental PI3K and pAkt [189], suggesting that the PI3K/Akt pathway in PE may be modulated by an array of factors.

MiR-126 has also been the subject of clinical studies; an initial study by Yan et al. [163] found a decrease in miR-126 levels in the placentas of PE patients as well as an upregulation of PIK3R2 and downregulation of PI3K, Akt, and pAkt mRNA and protein levels. In agreement, Khaliq et al. [190] reported lower protein levels of placental PI3K, Akt, and pAkt in preeclamptic placentas and a significant negative correlation between the proteins and several serum miRNAs, miR-222, miR-29a, and miR-181a, providing supporting evidence of a link between the PI3K/Akt pathway and miRNAs in PE. Reduced pAkt was seen in a further two studies in PE patients, which showed inhibition of PI3K/Akt in HUVECs stimulated the release of the anti-angiogenic factor soluble endoglin (sEng) [191,192]. One of the studies identified an association between lower pAKT and elevated circulating sEng in patients [191]. Overall, these findings suggest reduced activation of the PI3K/Akt pathway may contribute to the pathology of PE (Figure 2).

However, in placental explants and primary trophoblasts, inhibition of PI3K/Akt did not induce release of sEng [192]. Furthermore, in a trophoblastic JEG-3 cell line, inhibition of PI3K/Akt led to decreased expression of the anti-angiogenic factor sFLT-1 and increased or unchanged expression of the angiogenic factors, VEGF and placental growth factor [193]. This evidence suggests the PI3K/Akt pathway may exhibit cell-dependent effects, with its aberrant expression contributing to either pathological or protective processes depending on cell type. In contrast with the findings of Yan et al. [163], an upregulation of PI3K and Akt has been reported in the placentas of severe preeclamptic patients [193], indicating that, in addition to cell-dependent effects, genetic diversity in PE may underlie differences in PI3K/Akt pathway regulation [193]. One study observed a downregulation of Akt only in preeclamptic patients delivering before 34 weeks but not after [194], and another reported no difference in Akt levels compared with healthy controls [195]. It should be noted though, basal state was measured, and differences may only occur when Akt is stimulated [195]. Overall, there is evidence to support that a reduction of miR-126 in PE leading to an increase in PIK3R2 and decrease in the levels of PI3K, pAkt, and Akt regulates angiogenic and inflammatory processes (Figure 2). Evidence contradicting this direction of expression of miR-126 and its gene targets demonstrates the genetic diversity of PE and again highlights the issue of targeting miR-126 as a treatment for PE, particularly given the cell-dependent effects of miR-126 and the PI3K/Akt pathway.

## MiR-148/152

The miR-148/152 family, consisting of miR-148a, miR-148b, and miR-152, plays an important role in regulating immune-related processes and is implicated in the development and progression of multiple types of cancer, autoimmune disorders, and chronic inflammatory diseases (reviewed by [196]). In their mature form, miRNA family members share a common seed sequence and hence target orthologous genes [196]. For example, DNA methyl‐transferase 1 (DNMT1) was experimentally validated as a target of miR-148a in gastric cancer cells [197], miR-148b in lung cancer cells [198], and miR-152 in ovarian [199] and liver cancer cells [200], in each of which overexpression of the miRNAs induced tumor suppressive effects. In turn, the studies reported a downregulation of miR-148/152 family members in the cancerous tissues and/or cells, coinciding with the consistently reported overexpression of DNMT1 in each cancer type [201–204]. In contrast, the miR-148/152 family also demonstrate oncogene capabilities, indicating the family of miRNAs can modulate targets and processes in a cell-specific manner. For example, miR-148a was found to be upregulated in patients with hepatocellular carcinoma [205], and *in vitro* experiments demonstrated its ability to inhibit a tumor suppressor gene and stimulate cell growth, viability, and migration [205]. In addition to cancerous tissue, evidence suggests that the miR-148/152 family is involved in regulating placental development during pregnancy [206–208]. In trophoblast cells, all three miRNA family members target human leukocyte antigen-G (HLA-G) [206–208]. HLA-G plays an essential immunosuppressive role in mediating the maternal immune tolerance to the fetus by acting as an inhibitory ligand of maternal natural killer cells [209]. It is therefore postulated that reduced expression of miR-148/152 family members in the placenta in a healthy pregnancy may confer a protective effect by permitting sufficient HLA-G expression [206,208]. Correspondingly, evidence from preeclamptic animal models and patients points toward an upregulation of miR-148/152 family members inducing dysregulation of target genes and contributing to the pathogenesis of PE.

The role of miR-148/152 in PE has been investigated in two preeclamptic animal model studies in both of which Sprague Dawley rats were administered L-NAME [42,210]. Yang et al. [42] found miR-148a and miR-152 were significantly upregulated in the placentas of preeclamptic rats, which showed a significant increase in blood pressure and urinary protein as well as pathological placental changes, although no significant difference in fetal weight was observed. Zhang et al. [210] also observed that miR-152 was significantly upregulated, though it should be noted that a PE phenotype was not validated. In terms of miR-152 in preeclamptic patients, two studies reported significantly increased expression in the placenta [19,25], and one study reported increased expression in serum [62] (Table 3). For miR-148a, one study found an upregulation [26] and one study a downregulation [211] in preeclamptic placentas (Table 3). These studies were conducted in the chorionic plate and decidua-derived mesenchymal stem cells respectively, raising the possibility that dysregulation of miR-148a in PE may differ between placental cell types and/or layers, modulate different gene targets, and therefore contribute to pathological processes and/or confer protective effects. Although members of the miR-148/152 family share a common seed sequence, differences in their non-seed sequence may account for targeting of different mRNAs [196]. Hence, the observed upregulation of miR-152 in mild and severe patients and different areas of the placenta, as well as in animal models of PE, may indicate inhibition of miR-152 as a potential therapeutic strategy for PE. However, additional studies across PE subpopulations and placental layers are required to validate miR-152 as an upregulated placental miRNA in PE.

**Table 3:** Studies demonstrating significant change in miR-148/152 expression in PE patients compared with healthy pregnant women

Study	Type of study	Type of PE	Time of collection	Tissue sample	Direction of change
**miR-148a**					
Zhao et al. 2014 [211]	Microarray	Severe LO	Delivery	Decidua derived MSCs	Down
Zhou et al. 2016 [26]	RNA seq and qPCR	Diagnosed	Delivery	Chorionic plate from quadrant and central portion in placenta disc	Up
**miR-152**					
Zhu et al. 2009 [19]	Microarray and qPCR	Mild and severe	Delivery	Placenta villi	Up
Ura et al. 2014 [62]	Microarray	Severe	12–14 weeks	Serum	Up
Jiang et al. 2015 [25]	Microarray	Severe	Delivery	8 maternal and 8 fetal sites	Up

LO, late onset; MSCs, mesenchymal stem cells; Time of collection designated ‘delivery’ if not stated in study.

## Gene targets of MiR-148/152

**Figure 3:**
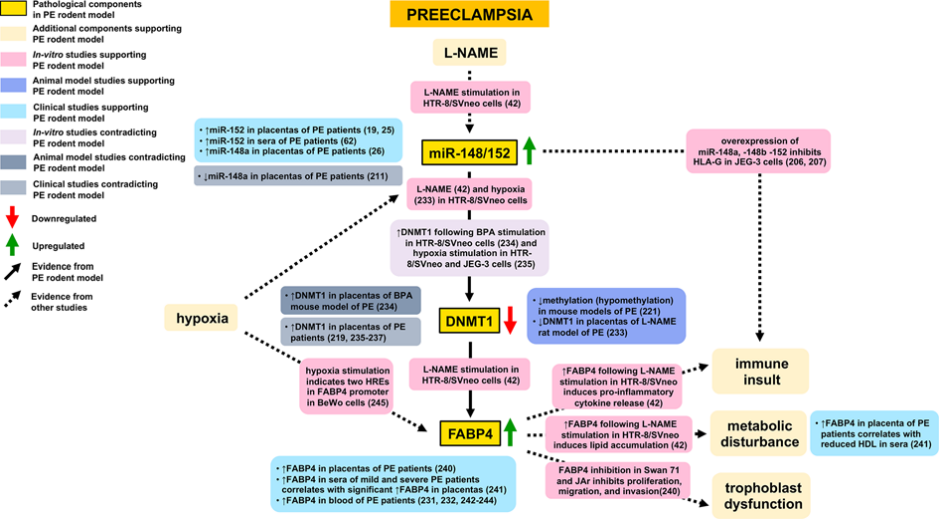
Supporting and contradicting evidence of proposed genetic components and pathological processes involved in PE based on PE rodent model study investigating the role of the placental miR-148/152 family miR-148/152, microRNA 148/152; miR-148, microRNA 148; miR-152, microRNA 152; DNMT1, DNA-methyltransferase 1; FABP4, fatty acid-binding protein 4; L-NAME, N (gamma)-nitro-L-arginine methyl ester; BPA, Bisphenol A; HREs, hypoxia response elements; HLA-G, human leukocyte antigen-G; HDL, high density lipoprotein.

The study investigating miR-148/152 in the L-NAME preeclamptic rat model that provides evidence of a PE phenotype also examined the expression of DNMT1, a gene target of miR-148/152, and fatty acid-binding protein 4 (FABP4), a gene target of DNMT1 [42]. Trophoblast cells incubated with L-NAME stimulated miR-148/152 and inhibited DNMT1 expression [42]. Reduced DNMT1 expression was associated with hypomethylation of FABP4 and thus increased FABP4 expression [42]. In line with these findings, a significant decrease in DNMT1 and increase in FABP4 mRNA and protein was observed in the placentas of L-NAME-induced PE rats and higher protein levels of FABP4 were seen in their sera [42]. DNMT1 belongs to the family of DNA methyltransferase enzymes that establish and maintain DNA methylation patterns [212]. DNA methylation is a major epigenetic mechanism involved in regulating gene expression. DNMT1 catalyses the addition of methyl groups to CpG islands located in the promoter region of a number of genes [212]. This prevents binding of transcription factors to the promoter and therefore primarily leads to silencing of gene expression [212]. Altered DNA methylation in the placentas of PE patients has been investigated extensively. Studies have examined its potential as a biomarker for PE [213] and the association between DNA methylation with clinical manifestations of PE [214]. Moreover, novel genes and miRNAs with differentially methylated regions in PE have been identified, and genes and miRNAs previously detected as differentially expressed in PE have been validated as differentially methylated [215–221]. Furthermore, an *in vivo* study investigating two mouse models of PE found that elevating placental adenosine caused placental DNA hypomethylation and produced a PE phenotype [222]. When placental adenosine was normalized to physiological levels, placental DNA methylation was restored and ameliorated the PE phenotype [222]. Collectively, these studies support a key involvement of DNA methylation and dysregulation of genes and miRNAs in the pathology of PE. One such target may be FABP4, which belongs to the family of fatty acid binding proteins that play a role in regulating lipid trafficking and cellular responses to lipids [223]. FABP4 is predominantly expressed in adipocytes and macrophages, in turn mediating inflammatory and metabolic pathways, such as regulating lipolysis and acting as a fatty acid chaperone [223]. High levels of circulating FABP4 have been identified in obesity [224], Type 2 diabetes [225], insulin resistance [226], hypertension [227], and atherosclerosis [228] as well as in gestational diabetes [229], gestational hypertension [230], and PE [231,232]. Hence, the miR-148/152 family may contribute to PE via modulation of DNA methylation patterns that leads to aberrant expression of downstream targets involved in metabolic and immune pathways (Figure 3).

In agreement with the findings by Yang et al. [42], a separate study in L-NAME-induced PE rats also reported a downregulation of DNMT1 in placentas and in hypoxia-treated trophoblasts [233]. In contrast, a study examining PE-like mice exposed to Bisphenol A (BPA) found a significant elevation in DNMT1 placental protein levels and an increase in DNMT1 expression in HTR-8/SVneo trophoblast cells exposed to BPA [234]. HTR-8/SVneo and JEG3 cells exposed to hypoxia also exhibited increased DNMT1 mRNA and protein levels [235]. An overexpression of DNMT1 is similarly seen in several clinical studies in preeclamptic placentas [235–237]. A separate study stratified preeclamptic patients into early onset (EO) and late onset (LO)PE detected significantly higher DNMT1 expression only in EO placentas [219]. Furthermore, a study that conducted DNA methylation analysis on candidate genes in preeclamptic placentas identified two CPG sites within DMNT1, one which differed between EOPE and LOPE patients as compared with a control group and one that only differed between EOPE and controls [238]. This suggests that differences in DMNT1 expression may be attributed to diverse PE genotypes. As previously identified, differences in the direction of expression of miR-148/152 family members may account for differences in the expression of DNMT1. Moreover, given the evidence of hypoxia and BPA exposure inducing DNMT1 expression in trophoblast cells, other factors may override the inhibitory effects of the miR-148/152 family on DNMT1 expression. In line with this, hypoxia-induced upregulation of DNMT1 in cardiac fibroblast cells was shown to be regulated by the transcription factor, HIF-1α [239]. Nonetheless, certain evidence suggests the miR-148/152 family may contribute to the development of PE by modulating DNA methylation (Figure 3).

Concerning FABP4, a significant increase in mRNA and protein levels was seen in L-NAME- treated HTR-8/SVneo trophoblast cells as well as in the placentas of L-NAME-induced PE rats [42]. Correspondingly, FABP4 DNA methylation levels were significantly reduced in L-NAME-treated trophoblast cells as well as in the placentas of preeclamptic patients [42]. Elevated FABP4 expression in L-NAME-treated trophoblasts led to accumulation of lipids and release of pro-inflammatory cytokines [42]. A separate study also reported significantly higher FABP4 mRNA and protein levels in the placentas of PE women [240]. It was found that inhibition of FABP4 in two trophoblast cell lines (Swan 71 and JAr) inhibited the proliferation, migration, and invasion of trophoblasts, indicating a key role for FABP4 in placental development [240]. Another study observed a significant increase in FABP4 levels in placentas from mild and severe PE patients, showing a correlation with elevated FABP4 and reduced high density lipoprotein in the sera [241]. Multiple studies have found higher circulating levels of FABP4, from as early as the first prenatal visit [242] to 8–13 and 24–48 weeks [243], 14 and 26 weeks [244], and the third trimester [231,232]. An *in vitro* study in BeWo trophoblast cells exposed to hypoxia validated two HREs in in the promoter of FABP4 [245]. This suggests that, in addition to the miR-148/152 family inhibiting DNMT1 and reducing methylation of FABP4, other factors may contribute to the elevated FABP4 levels seen in PE patients (Figure 3).

## MiRNAs as therapeutic targets

The number of miRNA-based therapeutics in clinical trials has steadily been increasing over the past decade, with phase I and II trials currently underway [246]. While the majority of miRNA therapeutics under development are for the treatment of cancer, researchers have been investigating their utility for viral infections and neurological, metabolic, inflammatory, and cardiovascular disorders [247]. Strategies to target dysregulated miRNAs are inhibitors (traditionally anti-miRNA oligonucleotides known as antagomirs that are complementary to the target miRNA) or mimics (primarily chemically synthesized double stranded RNA molecules) [248]. Use of these hydrophilic agents however carries disadvantages in terms of nuclease degradation, off-target binding, and poor cell membrane penetration [249]. In turn, a number of strategies have been introduced to enhance the efficiency and specificity of inhibiting or restoring miRNAs, including locked nucleic acid anti-miRs and miR-sponges and miRNA vectors and small molecules, respectively [248]. Furthermore, novel delivery systems using peptides and lipid nanoparticles have been developed to provide further protection against nucleases, prevent immune attacks, and improve targeted delivery [248]. The unique ability of miRNAs to target multiple genes and in turn specific components of a biological pathway or even entire or multiple biological pathways underlies their potential as therapeutic targets. For example, MGN-9103, an inhibitor of the cardiac specific miR-208a, has shown to prevent pathological cardiac remodeling in a heart-failure rat model, leading to improved cardiac function, overall health, and survival [250]. Microarray analysis revealed 131 genes were significantly differentially expressed in the hearts of treatment mice, of which 31 were identified as predicted gene targets of miR-208a [250]. Of the predicted targets, 28 genes were significantly increased, corresponding with an inhibition of miR-208a permitting an upregulation of otherwise inhibited target genes [250]. Although these findings demonstrate the therapeutic benefit of miRNAs’ pleiotropic nature, off-target effects and unintended toxicity are a key concern among miRNA-based therapeutics. Identifying a suitable miRNA target and developing efficient delivery systems to achieve therapeutically relevant outcomes therefore remain essential and represent major hurdles in developing clinically translatable miRNA-based therapeutics. These challenges are mirrored in research seeking to target altered placental miRNAs in pregnancy disorders.

Given the heterogeneous nature of PE, it is unlikely that a single placental miRNA will present itself as a suitable therapeutic target. Studies continue to investigate the utility of stratifying preeclamptic patients into distinct subtypes based on biomarkers, genetics, pathology, and/or clinical phenotypes; however, patients often present with overlapping elements and intermediate phenotypes [180,251–253]. In turn, the International Society for the Study of Hypertension in Pregnancy guidelines recommend classifying PE as with or without severe features or as superimposed (when pre-eclampsia develops in women with pre-existing chronic hypertension) and restricting the use of ‘mild’, ‘severe’, ‘EO’, and ‘LO’ to research [3]. These classifications highlight the diverse disease presentation seen among PE patients. Furthermore, microarray and next-generation sequencing studies in the blood [254] and placentas [255–257] of patients with different PE subtypes demonstrate not only common but also distinct gene expression profiles. Genetic heterogeneity among PE subtypes is similarly evident in terms of placental miRNAs [19,70,183,255,258,259] and partially explains inconsistencies in their reported direction of expression as previously discussed. However, it remains to be seen whether a differentially expressed placental miRNA may be restricted to specific subtype and therefore represent a precision medicine approach to targeting miRNAs in PE. In support of this approach are the increasing number of studies that show altered miRNAs in the blood early in gestation in preeclamptic patients correspond with differentially expressed miRNAs in preeclamptic placentas at term [260–263] and can therefore act as non-invasive biomarkers, with a recent study demonstrating their utility as biomarkers for PE subtypes [21]. A dysregulated blood-based miRNA in a subset of PE patients corresponding with altered expression in the placenta and therefore representing a therapeutic target conceptually illustrates an ideal scenario. However, altered miRNA expression profiles in PE subtypes both in maternal blood and the placenta still require validation in larger cohorts and replication in independent cohorts, presenting a challenge given the difficulty of recruiting pregnant patients for clinical samples. Hence, a suitable miRNA target for the treatment of preeclampsia, likely restricted to a subset of patients, remains to be identified.

In the previously mentioned study by Yan et al. [41], rats administered L-NAME showed significantly higher SBP and urinary protein levels compared with controls, providing evidence of a PE phenotype. Treatment rats went on to receive an intra-placental injection of Cy3-labeled agomiR-126, a miR-126 mimic [41]. This led to a 4.2-fold increase in miR-126 expression in treatment rats compared with non-treated PE rats [41]. In the placentas of the treatment rats, patches of miR-126 were detected by fluorescence microscopy, which were notably absent from PE and control rats [41]. These findings highlight the potential of intraplacental delivery of miRNA mimics for targeting the placenta, but investigation of miR-126 expression across other tissues would be required to confirm placenta-specific delivery. MiR-126 treated rats had significantly higher pup and placenta weights, enhanced microvessel density, and greater proportion of live pups [41]. Although there was a reduction in SBP by 15% in miR-126 treated rat compared with PE rats, this was not significant, and urinary protein levels were not reported. These findings support a key role for miR-126 in contributing to the clinical manifestations of PE even though clinically relevant therapeutic outcomes, in terms of blood pressure and urinary protein levels, were not achieved. Based on clinical studies reporting both up- and downregulation of miR-126, it is unlikely that miR-126 represents a suitable therapeutic target for all PE patients. This further supports the concept that altered miRNAs may underlie unique signatures of PE subtypes and identifying these should be the focus of future clinical studies. Accordingly, it may be more valuable to assess the therapeutic effect of targeting miRNAs in animal models of specific PE subtypes (e.g. EO, LO, mild, severe, and superimposed). Finally, with cell-dependent expression of miR-126 potentially contributing to inconsistencies in its direction of expression, targeting at the cellular versus tissue level may be essential for achieving therapeutically relevant outcomes. Both viral [264] and non-viral [265] delivery systems seeking to attain cell-specific targeting of miRNAs are under development but remain unexplored in the placenta *in vivo*, a potential novel avenue for research.

Currently under investigation is tissue-level targeting of placental miRNAs, which in itself represents a challenge. A recent study by Beards et al. [266] examined the feasibility of placental homing peptide-miRNA inhibitor conjugates as a therapeutic strategy for pregnancy disorders. A non-targeting miRNA inhibitor was administered to pregnant C57/BL6J mice intravenously to demonstrate proof-of-concept and assess safety parameters [266]. The inhibitor was detected primarily in the junctional zone, with lower levels evident in the labyrinth and decidua, and none was detected in vehicle control mice [266]. The miRNA inhibitor was not seen in any fetal organs examined but was detected in maternal tissue, namely the heart, liver, kidney, and uterus [266]. The study noted the importance of these findings, indicating systemic delivery of a miRNA inhibitor may lead to off-target effects. No significant difference in median fetal and placental weights, fetal–placental weight ratio, litter size, or number of resorptions was observed though, suggesting the miRNA inhibitor had no serious adverse effects in pregnancy [266]. In the second part of the study, three sequences, namely a scrambled miRNA inhibitor, a miR-145 inhibitor, and a miR-675 inhibitor were conjugated to the peptide CCGKRK and injected intravenously into pregnant mice [266]. These miRNAs were selected since miR-145 and miR-675 have shown to regulate placental growth and function [267–270] and found to be dysregulated in the placenta of pregnancies complicated by PE and intra-uterine growth restriction [270,271]. Inhibition of miR-675 in treatment mice led to a significant reduction in placental miRNA expression and significant elevation in median placental weight compared with control mice [266]. Inhibition of miR-145 did not induce a significant change in placental miRNA expression and median placental weight [266]. This may have arisen due to normalization of miR-145 expression levels, highlighting the issue of targeting miRNAs for a therapeutically relevant period of time. Even though pregnancy has a defined timespan, modulating miRNAs for a sufficient period to achieve a therapeutic benefit is another aspect that must be considered. It should be noted though that both miRNA-inhibitors did cause a significant increase in median fetal weight, and there was no change in litter size or fetal resorptions, indicating the inhibitors were well-tolerated in pregnant mice [266]. While validation of placenta-specific targeting was not examined in the study, the group had previously evaluated the placental homing ability of the CGKRK peptide [272]. The 5[6]-carboxyfluorescein labeled peptide was not detected in the heart, brain, liver, spleen, or lungs, but was found in the kidney [272]. In the placenta, the peptide was detected in decidual spiral arteries and the labyrinth, specifically in the endothelium of unremodeled vessels and endovascular trophoblasts of remodeled vessels, while none was found in the junctional zone [272]. Collectively, these studies demonstrate the potential to target specific cellular compartments of the placenta but also highlight the difficulty of achieving tissue-specific delivery. While cell-specific targeting may represent an ultimate goal, efforts to achieve placental-specific targeting should not be dismissed, particularly given the limited number of *in vivo* studies examining placental miRNA targeting. Alongside the development of novel delivery systems targeting placental miRNAs, a growing understanding of the pathological role of placental miRNAs in PE underlies their emerging potential as therapeutic targets and as a new form of treatment for PE.

## Conclusion

Animal model studies offer essential insight into the molecular and functional mechanisms underlying PE as they permit investigations not possible in pregnant women. Three studies utilizing rodent models of PE explored the role of placental miRNAs, miR-210, miR-126, and miR-148/152 respectively, by examining expression of the miRNAs, their regulators, and gene targets. A comparison of these findings with evidence from clinical studies, other PE animal models, and *in vitro* studies have allowed us to present a comprehensive evaluation of the interaction of genetic components and pathological processes in the placenta that underlie PE as well as evaluate their suitability as therapeutic targets.

Inconsistencies in the direction of expression of miR-210, miR-126, and miR-148/152 across clinical studies highlight the genetic heterogeneity of PE. It is therefore unlikely that a single placental miRNA may represent a suitable target for all PE patients. Future studies should focus on elucidating altered blood-based and placental miRNA expression profiles in PE patient subtypes or identifying an altered placental miRNA characteristic of a subset of patients as this will permit undertaking a precision medicine approach to targeting potential therapeutic miRNAs. Investigations in animal models of PE may in turn benefit from utilizing specific PE subtype models to elucidate the role of placental miRNAs. Finally, establishing methods for targeting placental miRNAs as well as specific placental cell types remain cornerstone experiments before placental miRNAs may serve as therapeutic targets for a new form of treatment for PE.

## Abbreviations

Ang: Angiotensin
BPA: bisphenol A
CVD: cardiovascular disease
DNMT1: DNA-methyltransferase 1
EC: endothelial cell
EPC: endothelial progenitor cell
ERK: extracellular signal–regulated kinase
FABP4: fatty acid-binding protein 4
GATA3: GATA binding protein 3
HDL: high density lipoprotein
HIF-1: hypoxia-inducible factor 1
HLA-G: human leukocyte antigen-G
HRE: hypoxia response element
HUVECs: human umbilical vein endothelial cells
IL: interleukin
KO: knockout
L-NAME: N(gamma)-nitro-L-arginine methyl ester
LPS: lipopolysaccharide
mRNA: messenger RNA
miRNA: microRNA
MSC: mesenchymal stem cell
NF-κB: nuclear factor-κB
pAkt: phosphorylated Akt
PE: preeclampsia
PI3K: phosphatidylinositol 3-kinase
PKB: protein kinase B
P-PIC: poly I:C
SBP: systolic blood pressure
sEng: soluble endoglin
sFlt-1: soluble vascular endothelial growth factor receptor-1
SGA: small for gestational age
SHRSP: stroke-prone spontaneously hypertensive
SPRED1: sprouty related EVH1 domain containing 1
STAT6: signal transducer and activator of transcription 6
Th1: T-helper type 1
Th2: T-helper type 2
TLR3: toll-like receptor 3
TNF-α: tumour necrosis factor α
VEGF: vascular endothelial growth factor
WT: wild type
